# A Comprehensive Molecular Survey of *Babesia* spp. Genetic Diversity in Kazakhstan’s Cattle Populations

**DOI:** 10.3390/vetsci13080755

**Published:** 2026-07-29

**Authors:** Alexandr Ostrovskii, Anara Ryskeldina, Viktoriya Lucay, Ayan Dauletov, Rashid Karmaliyev, Kassym Mukanov, Alexandr Shevtsov, Assel Akhmetova

**Affiliations:** 1 National Center for Biotechnology, Astana 010000, Kazakhstan; aleksadr141000@gmail.com (A.O.);; 2 The Institute of Veterinary Medicine and Agrotechnology, Zhangir Khan West Kazakhstan Agrarian Technical University, Uralsk 090009, Kazakhstan

**Keywords:** *Babesia*, bovine babesiosis, Kazakhstan, nested PCR, phylogenetics, cytochrome b, spatial epidemiology, tick-borne disease

## Abstract

Bovine babesiosis is a tick-borne disease of major veterinary and economic importance, yet molecular epidemiological data from Kazakhstan remain limited. This study investigated the prevalence, distribution, and species composition of *Babesia* spp. in cattle across all 17 administrative regions of Kazakhstan. A total of 6455 cattle blood samples from 171 villages were screened by nested PCR targeting the cytochrome b (*cytb*) gene, and positive samples were characterized by sequencing and phylogenetic analysis. The overall positive rate for *Babesia* spp. were 3.87% (95% CI: 3.41–4.37%). Of 250 PCR-positive samples, 215 yielded high-quality sequences and were assigned to four species: *Babesia major* (31.2%), *B. occultans* (29.6%), *B. bigemina* (18.0%), and *B. bovis* (7.2%). Marked regional heterogeneity was observed, with *B. major* and *B. occultans* predominating across most regions, whereas *B. bigemina* was dominant in Atyrau. Turkistan was the only region where all four species co-circulated. First molecular evidence of *B. bovis* and *B. bigemina* was obtained for Atyrau, Akmola, and West Kazakhstan regions. These findings expand current knowledge of bovine babesiosis in Kazakhstan and reveal a species composition dominated by *B. major* and *B. occultans*, contrasting with the *B. bovis/B. bigemina*-dominated patterns commonly reported elsewhere. The detection of pathogenic *Babesia* species in previously unsurveyed regions highlights the need for continued molecular surveillance.

## Simple Summary

The current study aimed to address an important epidemiological question on the distribution and diversity of bovine babesiosis in Kazakhstan. A comprehensive set of 6455 cattle blood samples was tested for *Babesia* spp. using nested PCR targeting the *cytb* gene, with species assignment via *18S* rRNA, *cytb*, and *cox1* sequencing, and combined this with spatial analysis to map the geographic distribution of infections. Overall, 3.87% of the samples studied were positive for *Babesia* spp. across 17 administrative regions of Kazakhstan. Phylogenetic analysis of the positive samples revealed that *Babesia major* (31.2%), *Babesia occultans* (29.6%), and *Babesia bigemina* (18.0%), whereas only 7.2% of *Babesia bovis* were identified. Spatial analyses showed significant geographic heterogeneity in positivity rates of babesiosis across Kazakhstan. The application of nested PCR combined with Sanger sequencing in this study enabled the detection of babesiosis in regions where it had not previously been identified, underscoring the need for molecular monitoring of vector-borne pathogens in cattle.

## 1. Introduction

Babesiosis is a major tick-borne infection caused by protozoan parasites of the genus *Babesia* (phylum Apicomplexa, order Piroplasmida) [1]. *Babesia* spp. infect a wide range of domestic, wild, and occasionally human hosts [2,3,4]. Although most *Babesia* species primarily affect animals, several, including *B. microti*, *B. duncani*, and *B. divergens*, are zoonotic pathogens causing human babesiosis [5,6]. The expansion of the ranges of zoonotic *Babesia* species and their vectors underscores the importance of these infections within the One Health concept, as the epidemiology of babesiosis is shaped by interactions among domestic and wild animal populations, tick vectors, climatic factors, and human risk [5]. While the most common species for cattle include *Babesia bovis* and *Babesia bigemina*, *Babesia divergens*, *Babesia major Babesia occultans* [7,8], *B. bovis* and *B. bigemina* are considered the most pathogenic species, capable of causing severe clinical disease. Globally, bovine babesiosis remains one of the economically significant tick-borne infections in livestock production.

In Kazakhstan, five tick genera (*Ixodes*, *Hyalomma*, *Dermacentor*, *Rhipicephalus*, *Haemaphysalis*) play a key role in the eco-epidemiological cycle of vector-borne diseases [9,10,11,12]. The distribution of *Babesia* is closely linked to the ecological habitat of the tick vectors [10,13]. The worldwide spread of ticks and the increasing prevalence of tick-borne diseases (TBD) have accelerated in recent years, including Central Asian countries. Recent studies have reported on the prevalence of vector-borne pathogens, including *Babesia* species in Kyrgyzstan [14,15]; the Xinjiang Uygur Autonomous Region of China [16,17,18] and Southern Kazakhstan [9,19].

Movement of animals from *Babesia*-endemic regions may increase the risk of disease in babesiosis-free areas. In Kazakhstan, climatic conditions and geography, particularly in warmer Southern regions, together with animal husbandry and management systems, provide a suitable environment for the spread of tick vectors and associated TBDs [10]. *Babesia* parasites are not included in the compulsory monitoring of cattle or small ruminants in Kazakhstan, which complicates the timely detection and control of the disease spread.

Detection of active *Babesia* cases includes conventional microscopy techniques [20], serology, and molecular diagnostics. However, the sensitivity of the traditional microscopy and serology methods is limited in cases of low parasitemia [7]. Molecular diagnostic techniques such as polymerase chain reaction (PCR) and nested PCR (nPCR) are more effective and recommended in the early stage of infection [21,22]. The sensitivity and specificity of such methods are very high; however, selection of a specific PCR target gene is crucial [23]. Most research studies use 18S ribosomal RNA (rRNA) gene as a primary target for *Babesia* species detection [24,25]. The mitochondrial cytochrome b (*cytb*) and cytochrome c oxidase subunit I (*cox1*) genes have been widely used as molecular markers of *Babesia* spp. detection, species differentiation, population genetic studies, and phylogenetic analyses [22,25,26]. In particular, nested PCR approaches targeting *cytb* are highly sensitive for detecting *B. bovis* and *B. bigemina* and may be particularly useful when testing samples with low parasitemia [27].

Despite these advantages, many available molecular assays are species- or group-specific and therefore optimized for only a limited number of epidemiologically important *Babesia* species. Such assays are effective for targeted diagnostics but may underestimate the broader diversity of *Babesia* spp., especially in regions where the composition of circulating piroplasmid species remains insufficiently characterized. Conversely, broad-range 18S rRNA-based assays targeting *Babesia* and *Theileria* spp. can amplify multiple piroplasmid taxa and, in some cases, have been shown to produce cross-genera amplification with other apicomplexan parasites, including *Hepatozoon*, *Toxoplasma*, and *Hammondia*. This may complicate molecular screening in endemic areas where *Babesia* infections occur alongside other tick-borne hemoparasitic infections, making sequencing-based confirmation essential for accurate species identification [28,29].

Although the epidemiology of tick-borne diseases in Kazakhstan was studied in some endemic regions [9,19,23], data on the molecular distribution of *Babesia* spp. obtained using highly sensitive PCR-based methods remain limited across most regions of the country. Therefore, the aim of the present study was to investigate the molecular distribution and species composition of *Babesia* spp. in cattle across Kazakhstan using a newly developed *cytb*-targeted nested PCR assay.

## 2. Materials and Methods

### 2.1. Ethical Approval

The original blood sampling was approved by the Ethics Committee of the National Center for Biotechnology (Protocol No. 2, 4 April 2022), and cattle owners provided informed consent. The secondary molecular analysis of archived samples for *Babesia* spp. was approved by the Local Ethics Committee of the National Center for Biotechnology (Protocol No. 4, 24 October 2023).

### 2.2. Study Design

This was a cross-sectional observational survey; no experimental intervention, randomization, or control group was used, as the study aimed to describe the positivity, distribution, and species composition of *Babesia* spp. in the cattle population across Kazakhstan. One blood sample was collected per individual, with the experimental unit being a single animal. A formal a priori sample size calculation was not performed; the sample size was determined pragmatically, taking into account Kazakhstan’s large geographic area and the need for broad representation across all 17 regions and 171 villages to capture regional heterogeneity in bovine babesiosis. Villages and farms were selected by convenience based on accessibility, with additional emphasis on the South Kazakhstan region, given its previously documented history of bovine babesiosis. Accordingly, a greater proportion of samples was collected from the Jambyl, Kyzylorda, and Turkistan regions to ensure adequate representation of areas where bovine babesiosis had previously been reported. Within each selected farm or communal herd, adult cattle available at the time of sampling were selected for blood collection. Thus, animal selection within herds was based on availability rather than formal random sampling. As such, no formal randomization was applied to sample or farm selection, and potential confounders such as regional sampling intensity, interannual variation (2022 vs. 2023), and farm management practices were not formally controlled for.

This study was designed as an observational survey; therefore, no experimental group allocation and formal blinding to the sampled collection were applicable. However, during PCR amplification and sequencing steps, the regional/village origin of each sample was blinded during analysis, with this information linked to results only at the subsequent epidemiological data analysis stage, thereby minimizing the risk of assessment bias.

### 2.3. Sample Collection

Cattle blood samples were collected across 17 regions (districts and 171 villages) of Kazakhstan during July–August 2022 and 2023. In total, 6455 blood samples were obtained from individual animals kept within private farms and common herds using dipotassium EDTA vacuum systems. Due to the seasonal nature of grazing (from March to October), animals over three years of age were selected to cover at least two grazing seasons. Samples were transported to the laboratory within 48 h at 4–8 °C and immediately used for DNA extraction. Sex, breed, and weight were not recorded because they were not relevant to the study’s epidemiological objectives. A subset of the current dataset was previously reported by Kadyrova et al. (2024), in which samples were analyzed for molecular detection of *Anaplasma* spp. [23]. The present study represents a secondary molecular analysis of the archived samples for the detection and characterization of *Babesia* spp.

### 2.4. DNA Isolation

Blood samples (300 μL) were lysed with 900 μL of red blood cell buffer, followed by 5 min of incubation and 5 min of centrifugation at 12,200× *g* [23]. The pellet was resuspended in 100 μL of buffer (400 mM NaCl, 10 mM Tris-HCl, 2 mM EDTA), 20% SDS (40 μL), and 20 mg/mL proteinase K (10 μL), then incubated at 50 °C for 2 h. After adding the lysis solution, the samples were incubated at 60 °C for 10 min. Silicon dioxide was then added, and the samples were incubated, mixed, and centrifuged. The pellet was washed with guanidine-ethanol buffer, dried at 60 °C, and DNA was eluted in 100 μL of TE buffer. DNA concentration was measured using a NanoDrop One spectrophotometer (Thermo Fisher Scientific, Madison, WI, USA).

### 2.5. Nested PCR and Species Assignment

Molecular detection of *Babesia* spp. and primary taxonomic assignment of positive samples were performed using nested PCR amplification of a fragment of the mitochondrial *cytb* gene. Additional molecular evidence supporting species assignment was obtained by PCR amplification and Sanger sequencing of the 18S rRNA [30,31] and mitochondrial *cox1* genes.

For *B. major*, *B. bigemina*, and *B. occultans*, the internal 18S rRNA primer pair yielded specific amplification in a single-round PCR. In contrast, no amplification was obtained for *B. bovis* using this protocol. Therefore, *B. bovis* was confirmed using a published nested 18S rRNA assay [32,33], in which the first-round amplification and lower annealing temperature in the second round improved amplification of this species.

Primers targeting the *cytb* fragment were designed in-house for the present study. Primer candidates were selected from conserved regions shared among *Babesia* taxa represented in the reference panel analyzed, including *B. bovis*, *B. ovis*, and *Babesia* sp. Dunhuang, *Babesia* sp. Xinjiang isolate Kashi, *B. caballi*, *B. motasi*, *Babesia* sp. Coco, *B. bigemina*, *B. gibsoni*, *B. canis rossi*, *B. canis canis*, and *B. canis vogeli*. Primer regions were further prioritized when their 3′ terminal positions contained discriminatory mismatches against non-target piroplasmid sequences represented in the alignment, including *Theileria* spp. and *Cytauxzoon felis*. This strategy was intended to improve broad-range detection of *Babesia* spp. while reducing the risk of non-specific amplification from related hemoparasitic agents.

Primer sequences, assay purpose, template volume, and thermal cycling conditions are provided in Table 1. For *cox1* amplification, the two forward primers (Babesia_Cox1_475-f1 and Babesia_Cox1_475-f2) were combined in a single reaction as an equimolar mixture to broaden coverage across divergent *Babesia* taxa.

Each PCR was performed in a final volume of 25 µL. The reaction mixture contained 12.5 µL of BioMaster HS-Taq PCR-Special (2×) reaction buffer (cat. no. MH011-1020, Biolabmix, Novosibirsk, Russia), forward and reverse primers at a final concentration of 400 nM each, template DNA or first-round PCR product, and nuclease-free water to the final volume. For first-round amplification, 5 µL of extracted DNA was used, corresponding to 80–150 ng of DNA per reaction. For nested reactions, 3 µL of the first-round PCR product was used as the template for *cytb* amplification, whereas 2 µL of the first-round PCR product was used for 18S rRNA-based confirmation of *B. bovis*. Each amplification run included a no-template (negative) control to monitor for contamination and a positive control of previously confirmed *Babesia* DNA.

PCR amplifications were performed using a Mastercycler X50 thermal cycler (Eppendorf SE, Hamburg, Germany). Unless otherwise stated, the cycling program included an initial denaturation at 95 °C for 5 min, followed by the locus-specific cycling conditions shown in Table 1. Each cycle consisted of denaturation, primer annealing, and extension at 72 °C for the indicated duration. A final extension was performed at 72 °C for 5 min.

PCR products were analyzed by agarose gel electrophoresis and visualized under ultraviolet light. All *cytb*-positive PCR products were submitted for sequencing. Based on the *cytb* sequence analysis, representative isolates encompassing the different *cytb* genotypes and detected *Babesia* species were selected for additional amplification and sequencing of the 18S rRNA and *cox1* gene. Species assignment was based primarily on comparative analysis of *cytb* sequences and was additionally supported by comparative analysis of the 18S rRNA and *cox1* sequences where available.

### 2.6. DNA Sequencing and Phylogenetic Analysis

Sanger sequencing was used to determine the nucleotide sequences of the amplification products using BigDye Terminator v3.1 Cycle Sequencing Kit (Thermo Fisher Scientific, Vilnius, Lithuania). The reaction products were analyzed on an Applied automated genetic analyzer. Biosystems 3730xl DNA Analyzer (Applied Biosystems, Carlsbad, CA, USA). Primary processing of chromatograms and assembly of consensus sequences were performed using SeqMan v6.1 software (DNASTAR) [32].

To construct phylogenetic trees, all available nucleotide sequences of the *cytb*, *cox1*, and 18S rRNA genes corresponding to the *Babesia* species used were downloaded from the NCBI GenBank database. Multiple alignment of nucleotide sequences was performed using the ClustalW algorithm in MEGA software v12.1.2 [33]. Phylogenetic analysis was performed using the maximum likelihood (ML) method with the Kimura 2-parameter model of nucleotide substitution evolution, accounting for the discrete gamma distribution of substitution rate variability among sites (G) and the presence of invariant positions (I). Statistical support for phylogenetic tree nodes was assessed using adaptive bootstrap analysis. Visualization and final processing of phylogenetic trees were performed using MEGA v12.1.2 [33].

### 2.7. Spatial Analysis

Due to the heterogeneous sampling effort across 171 sampled areas (within 17 administrative regions), raw sample counts were converted to the proportions of positive *Babesia* samples. The generalized additive model (GAM) was used to estimate broad-scale spatial variation in infection probability while accounting for unequal sample sizes across locations using the package *mgcv* in R (v4.5.2) [34]. The response variable was defined as a binary outcome using the number of positive samples and the number of tested samples per location *cbind*(*number*_*of*_*positives*, *number*_*of*_*samples* − *number*_*of*_*positives*).

Spatial effects were modeled using a two-dimensional thin plate regression spline of geographic coordinates of the sampled 171 areas: *s* (Lon, Lat, k = 20). The smoothing parameter was estimated using restricted maximum-likelihood (REML). Model performance was assessed using deviance explained and adjusted R-squared.

Residual spatial autocorrelation was assessed using Moran’s I to determine whether the fitted model adequately captured the spatial structure of the data, using a spatial weights matrix (*lw*) derived from *k*-nearest-neighbor relationships based on geographic coordinates.

## 3. Results

### 3.1. Detection of Babesia spp. Using nPCR

**Figure 1:**
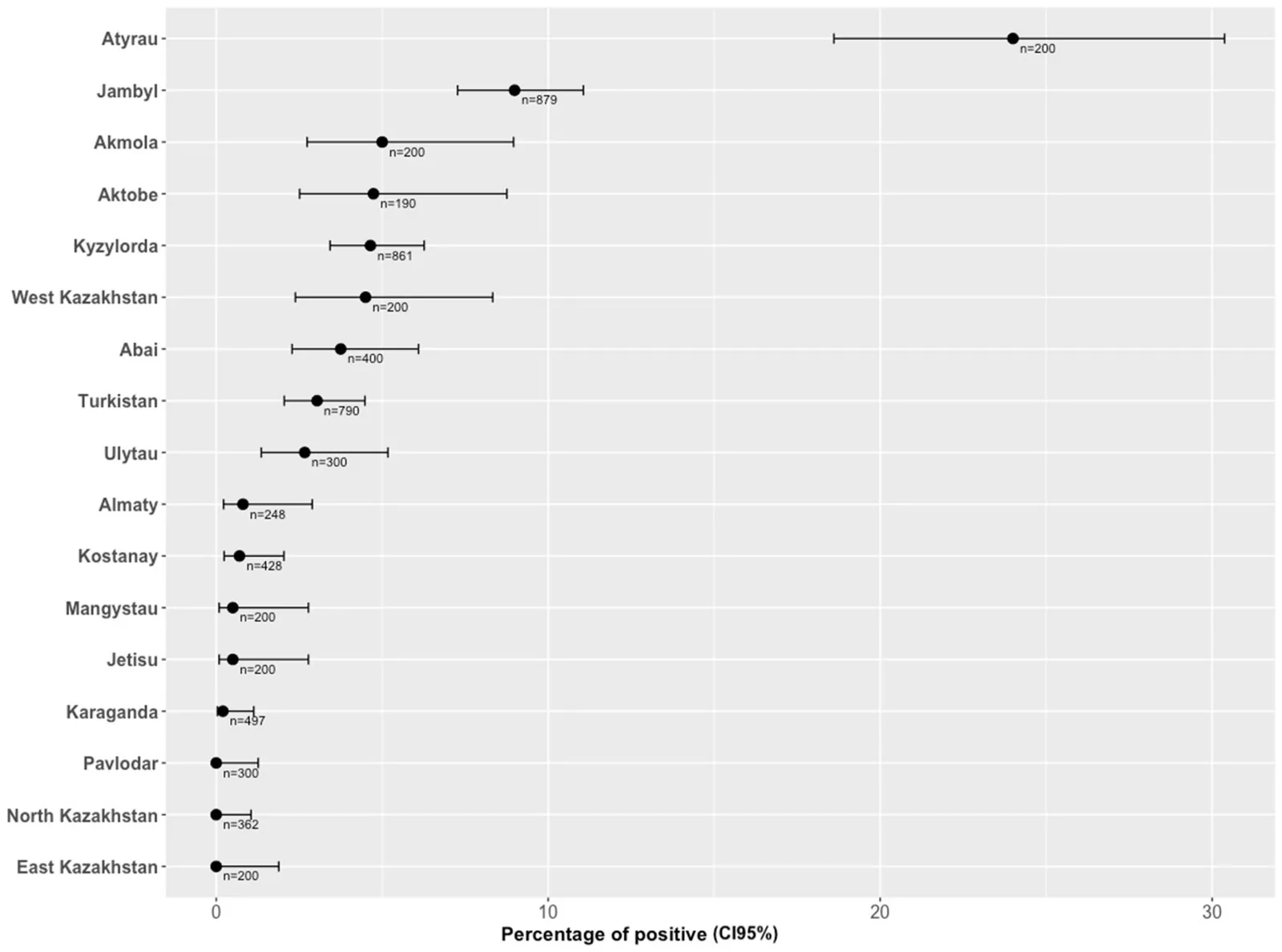
The percentage (CI95%) of *Babesia* spp. positive samples collected within 17 administrative regions of Kazakhstan, from the total sampled per region (shown as *n*).

Overall, the *cytb*-targeted nested PCR assay detected *Babesia* spp. in 250 of 6455 cattle blood samples, corresponding to a PCR positivity of 3.87% (95% CI: 3.41–4.37%). Regional PCR positivity is shown in Figure 1, whereas detailed information on the sampled regions and localities is provided in Supplementary Table S1.

**Figure 2:**
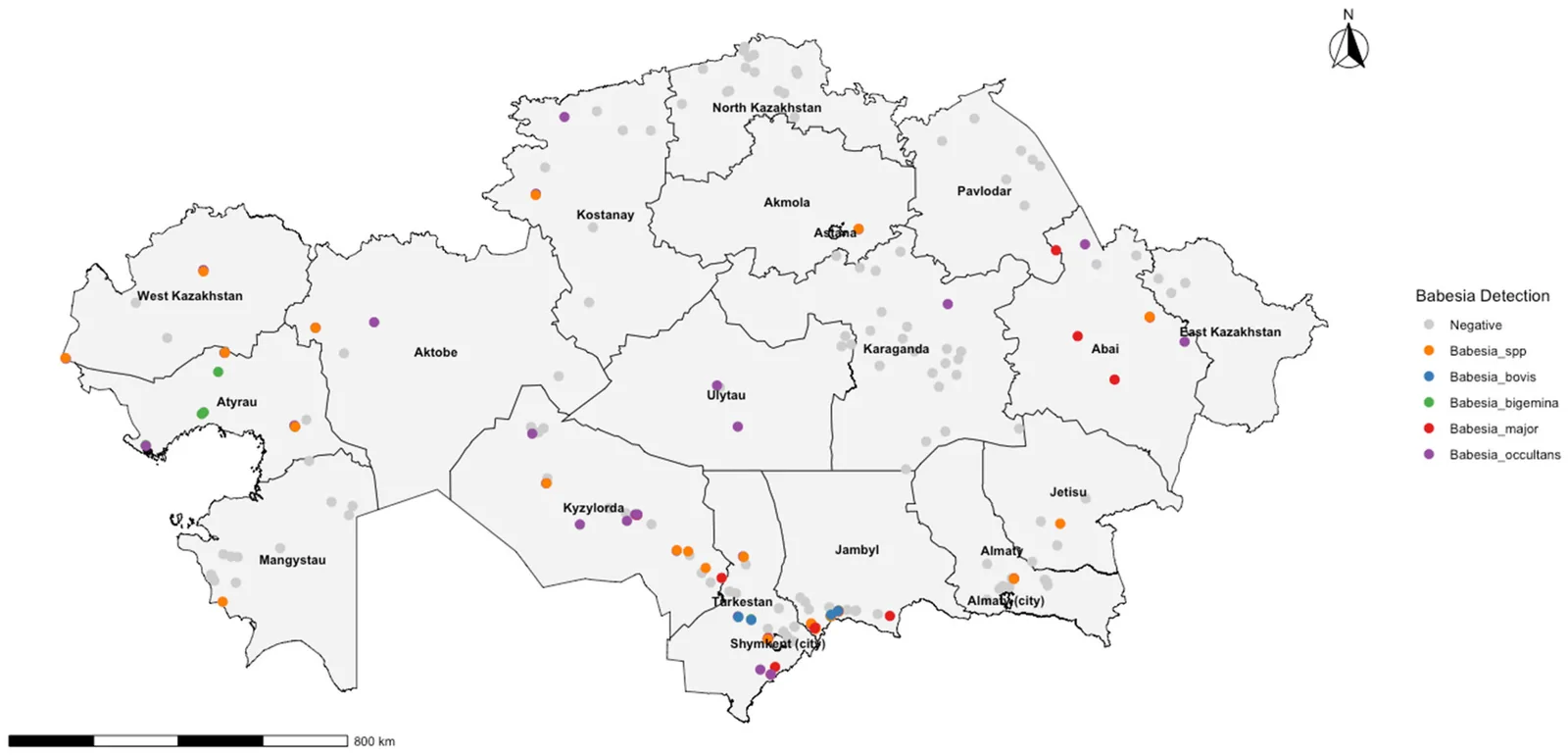
Spatial distribution of the cattle blood samples collected for *Babesia* spp. detection across 171 villages (17 administrative regions) in Kazakhstan. Positive samples for *Babesia* and species differentiation are shown in different colors. Each point represents a sampling village (locality) where at least one positive animal was detected. Multiple positive samples from the same village are represented by a single point. Where multiple localities shared identical coordinates after georeferencing, points were slightly jittered for visualization. The overlapping of points does not affect the scientific interpretation of the figure, as the spatial unit represented is the sampling locality rather than the individual animal or sample.

On average, 37.7 samples were collected per village (max *n* = 205, min *n* = 2). The spatial distribution of the 6455 collected blood samples for the detection of *Babesia* spp., which tested positive using nPCR method, is shown in Figure 2. Each point represents a sampling village where at least one positive animal was detected, rather than an individual positive animal. Because multiple positive samples could originate from the same village, point jittering was applied to improve visualization.

The largest proportions of collected samples originated from the Jambyl (13.6%), Kyzylorda (13.3%), and Turkistan (12.2%) regions. Samples positive for *Babesia* spp. varied considerably across regions, with the highest infection rate in Atyrau at 24% (*n* = 48 of 200 sampled), 9% in Jambyl (*n* = 79 of 879 sampled), and 5% in Akmola (*n* = 10 of 200 sampled) regions (Figure 1).

Previously, there was no data available on the prevalence of *Babesia* species in cattle samples, except in the Southern regions of Kazakhstan [9,19]. However, our findings are consistent with previous studies, the percentage of babesiosis positives in the south Jambyl (9%), Kyzylorda (4.6%), Turkistan (3.0%), Ulytau (2.7%), and Jetisu (0.5%) regions among tested animals in that region. Importantly, this is the first study to date showing the presence of babesiosis in Atyrau (24%), Akmola (5%), West Kazakhstan (4.5%), and Abai (3.8%) regions. According to the results, fewer positive cases were also found in Almaty (0.8%), Kostanay (0.7%), Mangystau (0.5%), and Karaganda (0.2%). There were no positives detected in North Kazakhstan Region, East Kazakhstan Region, or Pavlodar Region, which is most likely associated with the disease seasonality and colder weather in the North-East of Kazakhstan.

### 3.2. Babesia Species Differentiation and Phylogenetic Analysis

A total of 250 samples were positive by nested PCR. Of these, 215 (86.0%) yielded high-quality *cytb* consensus sequences suitable for downstream analyses, including BLASTn v2.17.0 comparison and phylogenetic reconstruction. The remaining 35 (14.0%) PCR-positive samples produced poor-quality Sanger chromatograms that precluded reliable sequence confirmation and were therefore excluded from all sequence-based analyses. Preliminary BLASTn searches of the low-quality chromatograms suggested similarity to *Babesia* spp. (92–97% identity); however, because of the poor sequence quality, these results were considered inconclusive and were not used for species identification or phylogenetic analyses.

All 215 high-quality *cytb* consensus sequences were compared with GenBank reference sequences using BLASTn. All sequences showed 100% query coverage, enabling reliable species identification. Sequences assigned to *B. bigemina* showed 99.24–99.43% nucleotide identity to the closest available reference sequences, whereas those assigned to *B. bovis* showed 98.47–98.66% identity. Corresponding *cytb* reference sequences for *B. major* and *B. occultans* were not available in GenBank, precluding direct sequence-based species assignment using *cytb* alone.

Initial phylogenetic reconstruction based on the *cytb* gene was performed using all 215 sequences obtained in this study. The resulting tree revealed a structured distribution of the Kazakhstani *Babesia* isolates into four major sequence clusters, comprising 32 distinct *cytb* genotypes in total (Supplementary Figure S1). These clusters differed markedly in size and internal diversity. One cluster included 74 isolates and showed the highest genotype diversity, with 22 *cytb* genotypes represented. A second cluster comprised 78 isolates grouped into six genotypes. The remaining two clusters were less heterogeneous and included 18 isolates represented by a single genotype and 45 isolates represented by three genotypes, respectively. This initial analysis demonstrated substantial *cytb* diversity among *Babesia*-positive cattle samples and provided the basis for subsequent genotype-based species assignment using reference sequences.

**Figure 3:**
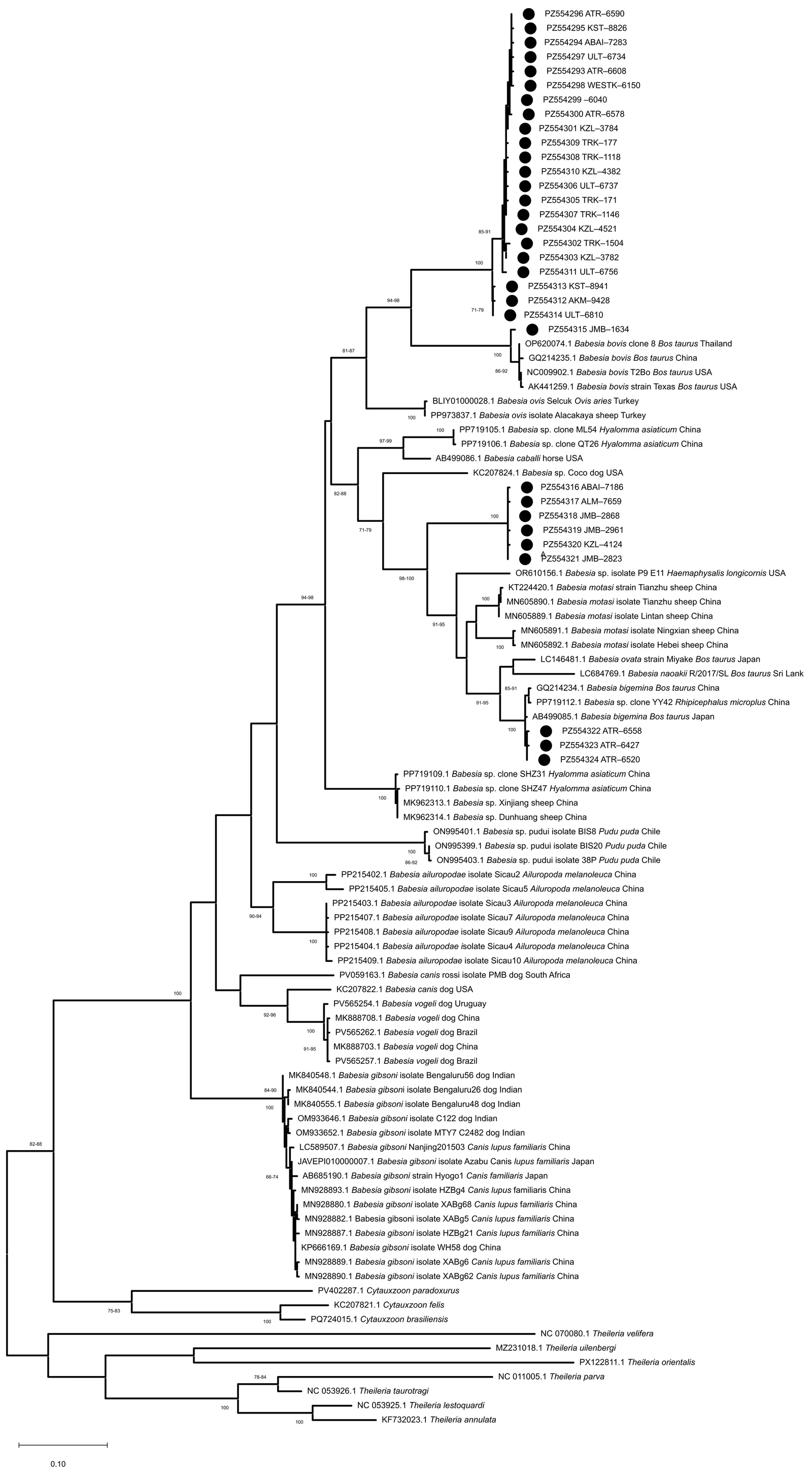
Genotype-based *cytb* phylogenetic tree of *Babesia* spp. The phylogenetic tree was constructed by the maximum likelihood method using a 499 bp fragment of the *cytb* (*cytochrome b*) gene and representative *cytb* genotypes identified among 215 sequenced *Babesia*-positive cattle samples. One representative sequence from each *cytb* genotype was included, together with reference sequences retrieved from GenBank. Bootstrap support values (%) are shown at the nodes. The scale bar represents the number of nucleotide substitutions per site. Sequences obtained in this study are marked with the (●) symbol.

**Figure 4:**
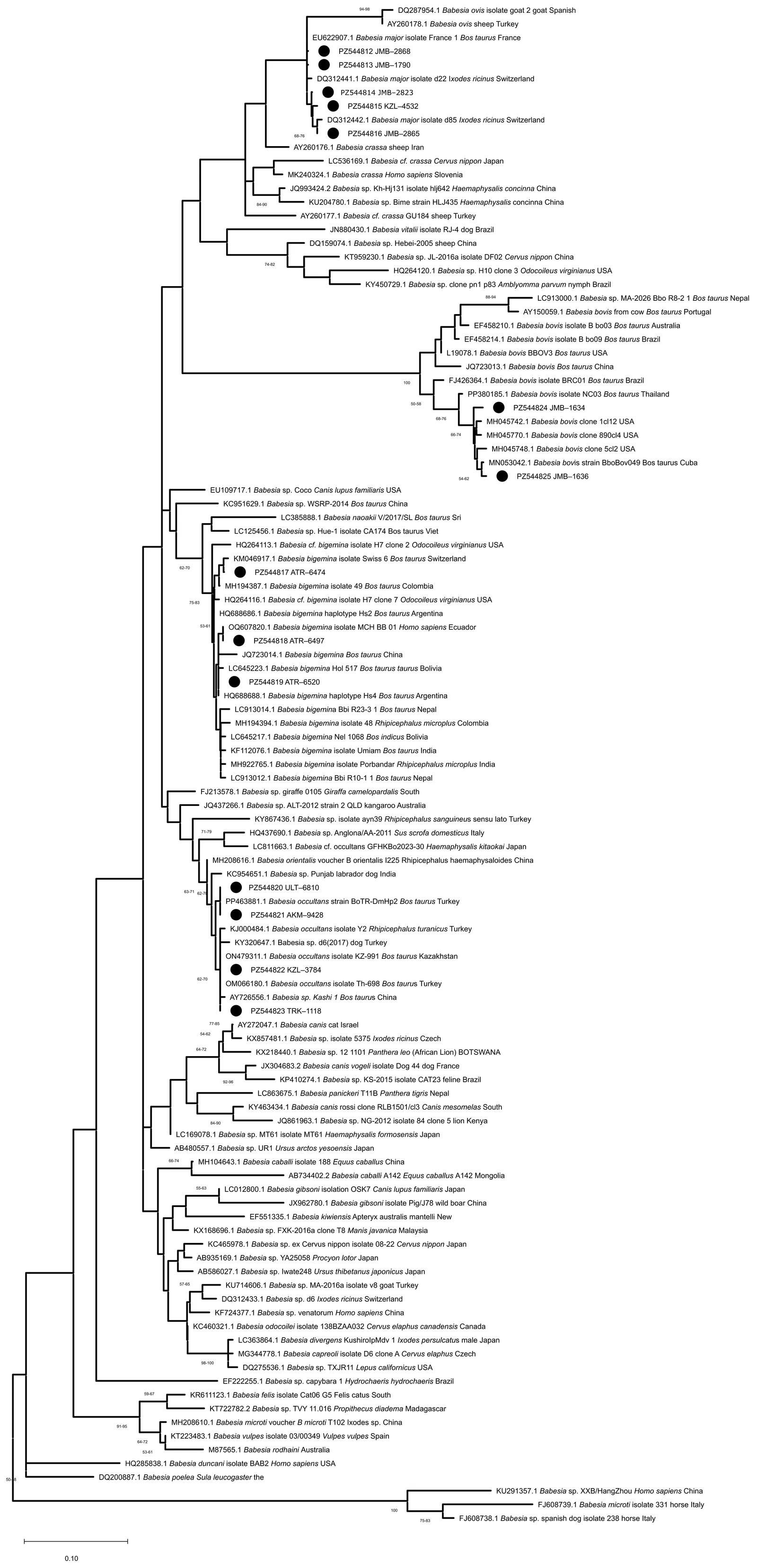
Phylogenetic tree of *Babesia* spp., constructed by the maximum likelihood (ML) method based on a 378 bp fragment of the 18S rRNA (18S ribosomal RNA gene) gene from cattle isolates using the MEGA 12 program. Bootstrap support values (%) are shown at the nodes of the phylogenetic tree. The scale bar reflects the number of nucleotide substitutions per site. Sequences obtained as part of this study are marked with the (●) symbol.

**Figure 5:**
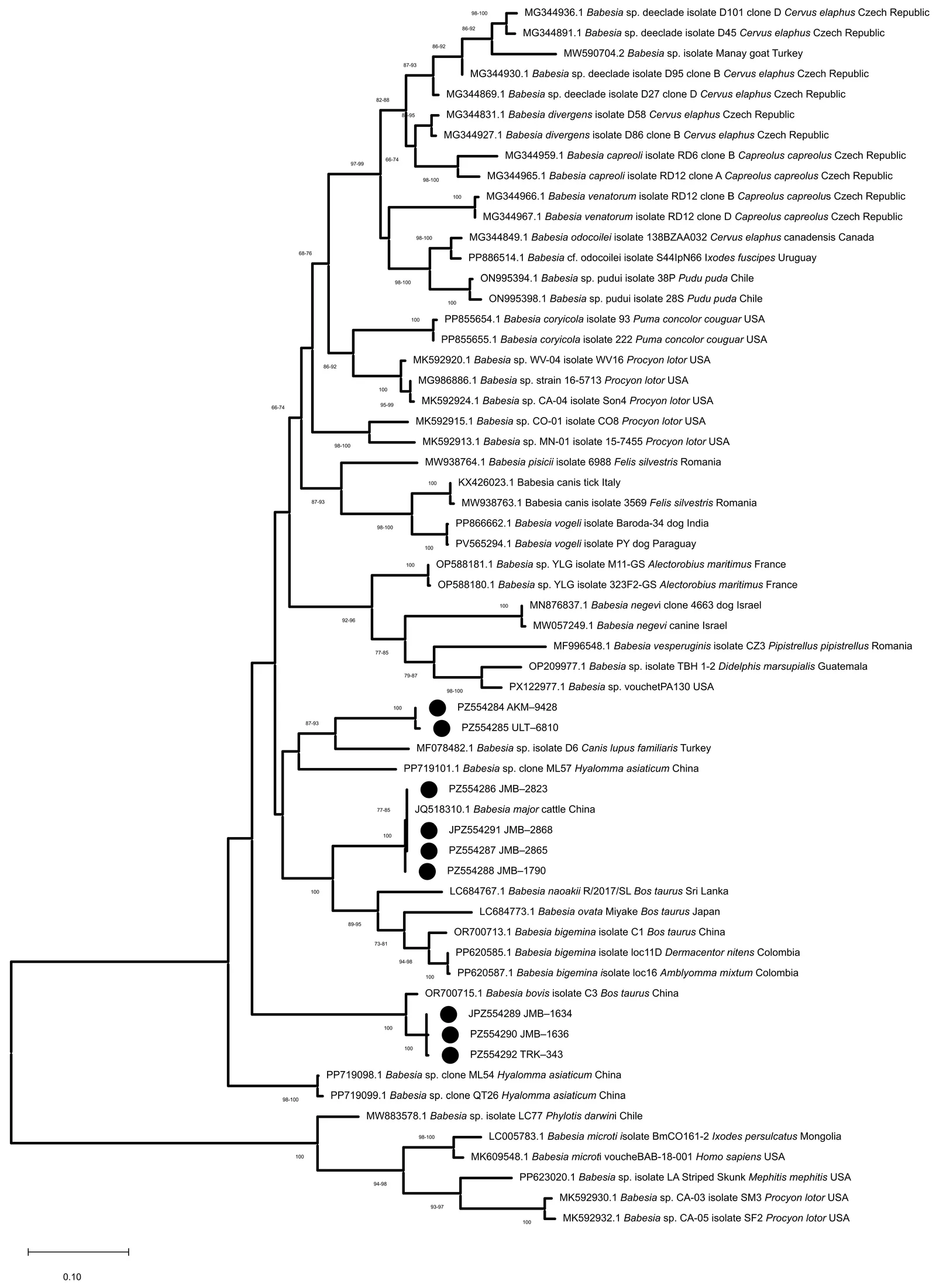
Phylogenetic tree of *Babesia* spp., constructed by the maximum likelihood method (ML) based on a fragment of the *cox1* (cytochrome *c* oxidase subunit I gene), 508 bp long, from bovine isolates using MEGA 12 software. Bootstrap support values (%) are shown at the nodes of the phylogenetic tree. The scale bar represents the number of nucleotide substitutions per site. Sequences obtained in this study are marked with the (●) symbol.

To avoid overloading the reference-based phylogenetic trees and to clarify the taxonomic position of the detected lineages, one representative sequence from each *cytb* genotype was selected for comparative phylogenetic analysis with GenBank reference sequences. The *cytb*-based reference tree supported the assignment of one genotype with *B. bovis* and three genotypes with *B. bigemina* (Figure 3). The remaining two divergent clusters, comprising 22 and six *cytb* genotypes, did not show sufficient resolution for confident species assignment based on *cytb* alone and were therefore further analyzed using additional genetic markers, namely 18S rRNA and *cox1*. Comparison with GenBank reference sequences supported the assignment of one *cytb* genotype to *Babesia bovis* and three *cytb* genotypes to *Babesia bigemina*. The *B. bovis*-assigned genotype showed 98.60% identity to the closest reference sequence, while the *B. bigemina*-assigned genotypes showed ≥99.4% identity. The GenBank accession numbers of all sequences generated in this study are provided in the corresponding phylogenetic trees (Figure 3, Figure 4 and Figure 5) and summarized in Supplementary Table S2.

High-quality 18S rRNA sequences were obtained for representative isolates of all four detected species, whereas *cox1* sequences were successfully generated for representatives of *B. major*, *B. occultans*, and *B. bovis*, but not for *B. bigemina*. Phylogenetic analysis of the 18S rRNA gene supported the species-level assignment of the *cytb* cluster comprising 74 isolates and *cytb* genotypes to the *Babesia occultans* lineage. Representative isolates from this cluster, including ULT-6810, AKM-9428, KZL-3784, and TRK-1118, grouped with *B. occultans* reference sequences (Figure 4). Compared with *cytb*, the 18S rRNA marker showed lower resolution of intra-cluster diversity within this group, supporting its utility for broader species-level placement rather than fine-scale discrimination of closely related *cytb* genotypes.

The *cytb* cluster comprising 78 isolates and six genotypes was represented in the 18S rRNA tree by several isolates, including JMB-2865 and JMB-1790, which clustered with reference sequences of *Babesia major*. This supported the assignment of this cluster to *B. major*. The 18S rRNA analysis also supported the placement of representatives of the cluster comprising 45 isolates and three *cytb* genotypes within the *B. bigemina* clade. In addition, representative isolates from the cluster comprising 18 isolates and a single *cytb* genotype grouped with *B. bovis* reference sequences, confirming the *cytb*-based assignment of this lineage to *B. bovis*. Overall, the 18S rRNA analysis corroborated the species-level interpretation of the four major *cytb* clusters, while showing lower discriminatory power than *cytb* for resolving genotype-level diversity within the *B. occultans*-related group.

Additional phylogenetic analysis based on the *cox1* gene was performed for a limited number of representative isolates. High-quality *cox1* sequences were obtained for representatives of the *B. occultans, B. major*, and *B. bovis* clusters, whereas *cox1* sequences of sufficient quality were not obtained for *B. bigemina* isolates. Therefore, assignment of the corresponding isolates to *B. bigemina* was based on *cytb* and 18S rRNA data. In the *cox1* tree, isolates JMB-2865 and JMB-1790 again clustered with *B. major*, confirming the species assignment inferred from the 18S rRNA marker. Representatives of the upper *cytb* cluster, including ULT-6810 and AKM-9428, formed a distinct lineage related to *B. occultans*; however, their exact placement was limited by the scarcity of available *B. occultans cox1* reference sequences in GenBank. Isolates assigned to the *B. bovis* cluster grouped within the *B. bovis* complex and occupied a separate branch, suggesting the presence of a genetically divergent *B. bovis* lineage circulating in Kazakhstan.

The combined analysis of *cytb*, 18S rRNA, and *cox1* supported the circulation of at least four *Babesia* species or species-level lineages in cattle populations of Kazakhstan: *B. major*, *B. occultans*, *B. bigemina*, and *B. bovis*. Among the 215 successfully identified isolates, *B. major* was the most frequently detected taxon, accounting for 78 isolates, followed by *B. occultans* with 74 isolates, *B. bigemina* with 45 isolates, and *B. bovis* with 18 isolates (Table 2, Figure 2). Importantly, these proportions reflect the distribution of sequenced *Babesia*-positive samples rather than direct estimates of clinical disease burden.

The geographical distribution of species-assigned isolates revealed marked regional differences in *Babesia* species composition across Kazakhstan. In the southern part of the country, Jambyl region showed the highest species diversity, with the detection of *B. major, B. bovis*, and *B. bigemina*. Kyzylorda and Abai regions were dominated by *B. major* and *B. occultans*, whereas Turkistan was the only region in which all four identified *Babesia* taxa were detected. In western Kazakhstan, the Atyrau region was characterized mainly by *B. bigemina*, with a smaller number of *B. occultans* infections. *Babesia occultans* was also detected in Akmola, Aktobe, Karaganda, Kostanay, Ulytau, and West Kazakhstan regions. Overall, these results indicate that the composition of circulating *Babesia* species differs substantially between regions, with pathogenic bovine species such as *B. bigemina* and *B. bovis* showing a more restricted but epidemiologically important distribution.

### 3.3. Spatial Analysis of Babesia Species

Spatial analysis of *Babesia* spp. samples collected across 171 villages in Kazakhstan revealed a structured pattern of positive cases using the generalized additive model (GAM). The model explained 31.9% of the total deviance, with an adjusted R^2^ of 0.164 (REML = 396.87; *n* = 171), indicating that a significant portion of the observed variation in positivity was explained by geographic location.

Assessment of model residuals showed no evidence of remaining spatial autocorrelation. Moran’s I, calculated from Pearson residuals, was negative and non-significant (Moran’s I = −0.05, *p* = 0.84). This indicates that the spatial structure in the data was adequately captured by the model and that residuals were spatially independent.

## 4. Discussion

This study constitutes the first nationwide molecular survey of *Babesia* spp. in cattle in Kazakhstan, comprising 6455 animals from 171 localities across all 17 administrative regions. The overall molecular positivity was 3.87% (95% CI: 3.41–4.37%), and four species were resolved using a combined *cytb*, 18S rRNA, and *cox1* marker strategy. The most biologically informative aspect of the present study was the species composition of the detected *Babesia* assemblage. *B. major* (31.2% of sequenced isolates) and *B. occultans* (29.6%) predominated, whereas the recognized pathogenic bovine species *B. bigemina* (18.0%) and *B. bovis* (7.2%) were detected at lower frequency. This profile departs from the *B. bovis*/*B. bigemina*-dominated pattern typical of many tropical and subtropical endemic settings, and instead describes a distinct Central Asian, steppe-associated pattern in which less virulent or usually subclinical cattle-associated *Babesia* species are prominent [9,19,20,35].

The detected molecular positivity rate of 3.87% indicates a relatively low prevalence of active *Babesia* infections in the sampled adult cattle population, although marked regional heterogeneity was observed. PCR detects circulating parasite DNA at the moment of sampling, and because serology was not performed, cumulative exposure cannot be inferred from these data alone. The strong method-dependence of *Babesia* estimates is well illustrated in Mongolia, where PCR-based detection of *B. bovis* and *B. bigemina* reached 27.9% and 23.6%, respectively, whereas a separate serological survey reported 18.0% and 22.4% seropositivity [36,37]. Conventional microscopy, still used in many routine veterinary settings, is less sensitive in chronic, low-parasitaemia carriers and cannot reliably differentiate morphologically similar *Babesia* species [19,20]. Sampling in the present study was restricted to July–August of 2022–2023 and to cattle older than three years, capturing a single seasonal window rather than the full annual transmission cycle. The nested *cytb* assay was designed to maximize broad-range detection across *Babesia* taxa and is therefore well suited to identifying low-parasitemia carriers that microscopy may miss.

These results extend and reframe work previously concentrated mainly in southern Kazakhstan. Kuibagarov et al. [19] examined adult cattle from Turkistan and Jambyl using PCR and reported an overall *Babesia* spp. prevalence of 24.7%, significantly higher in Turkistan (30.4%) than in Jambyl (13.5%); among 127 unambiguously typed positive samples, *B. occultans* was most frequent (43.3%), followed by *B. bigemina* (31.5%) and *B. major* (25.2%), with frequent *Theileria* spp. co-infection. The lower nationwide positivity rate observed in the present study is likely attributable to differences in study design and sampling framework. The earlier investigation was conducted as a targeted seasonal survey in recognized endemic foci, whereas the present study represents a country-wide screening effort encompassing ecologically diverse and previously unsurveyed regions. Tick-based studies support the plausibility of the detected species spectrum. Sang et al. [9] collected 6107 adult ticks in eastern and southern Kazakhstan. They detected piroplasm DNA in representative specimens, including *B. occultans* in *Dermacentor marginatus* from Saryagash, Turkistan. In contrast, a later survey of ticks from six regions detected two *Babesia* species, *B. occultans* and *B. caballi*, among 272 molecularly examined ticks [38]. Local microscopy-based reports have also recorded *B. bigemina* and *B. bovis* in southern Kazakhstan, including Kyzylorda and Turkistan, although such data constitute lower-level evidence than sequencing-confirmed molecular studies [39,40]. Together, these data indicate that the southern focus is one part of a broader, spatially heterogeneous national picture, and that the predominance of *B. major* and *B. occultans* in cattle is not confined to the two southernmost provinces.

The Atyrau Region had the highest molecular positivity rate recorded in the present study (24.0%, 48/200) and constitutes the first molecularly documented occurrence of *Babesia* spp. in cattle from western Kazakhstan. However, the lack of clinical, haematological, longitudinal, and concurrent tick data precludes interpretation of these findings as evidence of an outbreak and instead supports the designation of Atyrau as a priority region for follow-up surveillance. Independent, non-peer-reviewed monitoring from West Kazakhstan reported blood-parasite DNA in approximately 32% of cattle and up to 100% tick infestation in the steppe zone, with species-specific confirmation dominated by *Theileria annulata* and no detection of *B. bovis* [12]; because this source is a preprint, it is treated here only as preliminary contextual evidence. The distinguishing feature of Atyrau in the present dataset is the local predominance of *B. bigemina* (36 of 48 sequenced positive samples), an unusual pattern for Kazakhstan that warrants targeted confirmation through paired seasonal sampling of blood and ticks combined with species-specific qPCR or multiplex PCR, to determine whether the observed predominance of *B. bigemina* reflects stable local transmission, recent vector or livestock movement, or sampling bias. The western region is ecologically relevant in this respect, since *Hyalomma* and other ixodid ticks are documented in western Kazakhstan and Atyrau, and previous reviews indicate active circulation of tick-borne pathogens in this area [10,12].

The predominance of *B. major* and *B. occultans* is most parsimoniously explained by the structure of the local ixodid fauna rather than by climate alone. The major vectors of the most pathogenic bovine *Babesia* species, *B. bovis* and *B. bigemina*, are one-host *Rhipicephalus (Boophilus)* ticks, especially *R. microplus* in tropical and subtropical regions [20]. These ticks appear less central in Kazakhstan than *Hyalomma, Dermacentor*, and *Haemaphysalis*, which are widely reported in regional tick–faunal studies [10,11,38]. The observed species composition is consistent with established vector–parasite associations. *B. major* is classically associated with *Haemaphysalis punctata* [35], while *B. occultans* has been detected in *Hyalomma* ticks, with molecular evidence of transstadial and transovarial transmission in *Hyalomma marginatum* and *Rhipicephalus turanicus* in Turkey [41,42]. Nationwide and regional tick surveys are consistent with this interpretation, having reported *D. marginatus, Hyalomma asiaticum, H. anatolicum, H. scupense, H. marginatum, Ixodes ricinus, I. persulcatus*, and other ixodid species from livestock and regional collections in Kazakhstan [10,11,38]. The proven *B. bigemina* vector *Rhipicephalus annulatus* has also been recorded in Turkistan [43], but the overall restricted distribution and abundance of *Rhipicephalus (Boophilus)* vectors likely limit *B. bovis*/*B. bigemina* transmission over much of the country, while the sharply continental climate, with prolonged cold winters and a short vector season in northern and eastern regions, may further constrain the establishment of *Rhipicephalus*-dependent transmission cycles.

Set against neighboring and regional endemic settings, the Kazakhstani profile is distinctive, although comparisons must be made with caution because studies differ in diagnostic targets, host populations, seasons, and sampling designs. Within Central Asia, Kyrgyzstan exhibits marked heterogeneity, underscoring the local, method-dependent nature of piroplasmid communities rather than a single uniform pattern. The first molecular survey of Kyrgyz cattle detected three piroplasms—*Theileria orientalis* (most prevalent, 32.8%), *T. annulata*, and low-frequency *B. major* (1.3%)—with no *B. bovis* or *B. bigemina* recorded [15]. A subsequent PCR-and-sequencing survey of 319 cattle across five provinces broadened the detected community to eight vector-borne species, including *B. bovis*, *B. bigemina*, *B. major*, *B. occultans*, *T. annulata*, and *T. orientalis* [14]. Most recently, a molecular survey of 531 cattle reported *T. orientalis* (47.83%) and *T. annulata* (25.61%) as dominant, with *B. major* (0.19%) and *B. occultans* (0.38%) only marginally detected, and neither *B. bovis* nor *B. bigemina* found [44]. These divergent Kyrgyz datasets indicate that Central Asian piroplasmid communities are highly local and method-dependent. Comparable modern molecular data on cattle from Uzbekistan are limited. Historical non-molecular studies reported the occurrence of *B. bigemina* and *B. bovis* in cattle, suggesting a species composition more strongly associated with the classical pathogenic bovine *Babesia* spp. than that observed in Kazakhstan [45].

To the east and south, the contrast is clearer. In China, a 14-province nested-PCR survey of 646 cattle, dairy cattle, and yaks reported *B. bovis*, *B. bigemina*, and *B. ovata* at 20.7%, 9.3%, and 1.5%, respectively, with mixed infections in several provinces [46]. In Mongolia, PCR screening of 725 cattle from 16 provinces detected *B. bovis* (27.9%), *B. bigemina* (23.6%), and *Babesia* sp. Mymensingh (5.4%), and a separate serological survey confirmed broad exposure to *B. bovis* and *B. bigemina* [36,37]. In West Azerbaijan Province, Iran, PCR-positive cattle were dominated by *B. bigemina* (92.5% of *Babesia*-positive samples), with *B. bovis* detected less frequently (7.5%) [47]. Turkey has a broad bovine *Babesia* fauna, with *B. bovis*, *B. bigemina*, *B. divergens*, and *B. occultans* reported alongside competent vectors other than *R. microplus* [48]. For Russia and Siberia, cattle-level *Babesia* prevalence in territories bordering Kazakhstan remains insufficiently characterized; recent tick study detected *B. occultans*, *B. caballi*, and *T. equi* in *Dermacentor* spp. from Russian Siberia, but this cannot be translated directly into cattle prevalence [49]. Against this background, Kazakhstan occupies a transitional Eurasian position: *B. bovis* and *B. bigemina* are present and of veterinary significance, particularly in Atyrau, Jambyl, Akmola, and West Kazakhstan, yet the national sequenced dataset is dominated by *B. major* and *B. occultans*. This contrasts with the predominance of *B. bovis* and *B. bigemina* reported from several intensively studied eastern and southern endemic settings, and is most consistent with a steppe and semi-desert vector ecology in which *Hyalomma*, *Dermacentor*, and *Haemaphysalis* contribute more strongly to local piroplasmid transmission than *Rhipicephalus* (*Boophilus*) vectors [10,11,35,38,,42].

A clear distinction must be drawn between molecular detection and clinical babesiosis. Because systematic clinical examination, haematological evaluation, treatment history, and paired tick data were not available, the present results document *Babesia*-DNA-positive cattle and molecular evidence of infection or carriage, not clinical disease. Although *B. major* was the most frequently detected species in the sequenced subset, this should not be interpreted as indicating the greatest veterinary importance. The principal clinical concern remains the detection of the highly pathogenic bovine species *B. bigemina* and *B. bovis*, despite their lower frequency in the present study. Even so, the restricted detection of *B. bovis* and *B. bigemina* is epidemiologically important. WOAH describes *B. bovis* as generally more pathogenic than *B. bigemina* or *B. divergens*, with acute infection often characterized by fever, ataxia, anorexia, circulatory shock, and sometimes nervous signs, and with peripheral parasitemia that can remain below 1%, making microscopy insensitive [20]. The first molecular evidence of *B. bovis* and/or *B. bigemina* in previously unsurveyed regions, including Atyrau, Akmola, and West Kazakhstan, may reflect either recent changes in vector or livestock movement or long-standing under-detection owing to limited molecular surveillance—a concern that is particularly relevant for imported or pathogen-naive animals, in which primary infection later in life can precipitate severe clinical disease under conditions of unstable endemicity [19,35]. *B. occultans* should likewise not be dismissed as clinically irrelevant: although usually subclinical, it has been associated with clinical disease in cattle, including symptomatic cows in Iran [50]. These findings are consistent with the view that *B. major* and *B. occultans* are less virulent or usually subclinical, rather than strictly non-pathogenic. Taken together, these findings indicate that the *Babesia* population detected in Kazakhstan is dominated by species generally regarded as less virulent than *B. bovis* and *B. bigemina*.

The multi-marker design clarified the complementary value and limitations of each locus. The mitochondrial *cytb* marker provided high-resolution genotype-level discrimination, especially within the *B. occultans*-related cluster, supporting its use as the primary screening and typing target in this study. The nuclear 18S rRNA gene was more suitable for broad species-level placement than for fine intraspecific resolution, while *cox1* added resolution for selected lineages, including the *B. bovis*-related branch, but was constrained by limited reference coverage in GenBank; this limitation was most evident for *B. occultans*, and no high-quality *cox1* sequence was obtained for *B. bigemina* in the present dataset. The failure of 35 of 250 PCR-positive samples to yield interpretable Sanger chromatograms is plausibly attributable to low parasitemia, mixed infection, degraded archived DNA, or co-amplification of closely related taxa, which is a recognized limitation of Sanger-based typing in broad-range assays. Future studies should evaluate these samples using alternative primer sets or additional genetic markers to further assess potential non-specific amplification and improve confirmation rates.

The generalized additive model explained 31.9% of the deviance (adjusted R^2^ = 0.164), and Moran’s I on the residuals was non-significant (I = −0.05, *p* = 0.84), indicating that the broad geographic structure was adequately captured and that residuals were not detectably spatially autocorrelated. The spatial analysis complemented the descriptive distribution maps by accounting for heterogeneous sampling effort across the study area. Unlike visual inspection of sampling locations, the GAM estimates spatial variation in the probability of *Babesia* detection independent of the number of animals sampled at each location. The absence of significant spatial autocorrelation in the model residuals indicates that the fitted spatial surface adequately captured the broad-scale geographic structure of the data. Geography, therefore, accounts for a meaningful but partial share of the variation in molecular positivity, with the remaining variation most plausibly explained by local tick ecology, seasonality, breed and age structure, acaricide-treatment regimes, herd movement, irrigation, microclimate, and uneven sampling intensity.

Several limitations should be acknowledged. Sampling effort was uneven across regions, with fewer specimens from some eastern and western areas due to Kazakhstan’s vast territory and limited access to remote pastoral zones, which may have biased prevalence estimates and led to an underestimation of *Babesia* diversity in some ecological settings. Sampling covered only the mid-summer grazing period and did not include the full seasonal cycle. No concurrent tick collections were performed from the same animals or localities. Consequently, associations between the detected *Babesia* species and potential tick vectors remain inferential. Detection of *Babesia* DNA in ticks alone would not demonstrate vector competence, which requires evidence of pathogen acquisition, maintenance, and transmission by the vector. Clinical, haematological, treatment, breed, and acaricide-use metadata were not available, and the absence of serology prevents assessment of past exposure. Finally, Sanger sequencing resolves mixed infections less effectively than deep amplicon sequencing, and amplification of a predominant template does not exclude the simultaneous presence of additional *Babesia* species within the same sample. Furthermore, although species assignment was supported by analysis of the 18S rRNA and *cox1* loci for representative isolates, not all PCR-positive samples were characterized using all three genetic markers. Consequently, the detected taxa should be interpreted as molecular species assignments rather than definitive species identifications for every PCR-positive sample. Although the *cytb*-targeted nested PCR assay provided highly sensitive detection of *Babesia* DNA, species assignment was based primarily on *cytb* sequences and supported by additional markers only for representative isolates. Furthermore, Sanger sequencing of broad-range PCR products predominantly reflects the dominant template and therefore cannot exclude mixed infections or the presence of additional *Babesia* species at lower abundance within individual samples. Future studies combining seasonal, paired host–tick sampling with quantitative PCR, amplicon sequencing, vector-competence experiments, and economic-impact assessment would provide a substantially stronger epidemiological framework.

## 5. Conclusions

This nationwide molecular survey provides the first country-wide evidence of *Babesia* spp. infection in cattle in Kazakhstan, with an overall molecular positivity of 3.87% and four species identified across 17 administrative regions. The less virulent or usually subclinical cattle-associated species *B. major* and *B. occultans* predominated, a pattern that contrasts with the stronger *B. bovis*/*B. bigemina* signal reported from several intensively studied eastern and southern regional settings and is consistent with the distinctive steppe tick ecology of Kazakhstan. The recognized pathogenic species *B. bovis* and *B. bigemina* were nonetheless detected in several previously unsurveyed regions, including Atyrau, Akmola, and West Kazakhstan, identifying western Kazakhstan as a priority for follow-up surveillance. Collectively, these findings substantially expand current knowledge of bovine *Babesia* circulation in Kazakhstan and support the implementation of species-specific molecular surveillance and regionally targeted, vector-informed tick-control strategies.

## Figures and Tables

**Table 1:** Primers and amplification conditions used for molecular detection and species assignment of *Babesia* spp.

Target Gene	PCR Format/Round	Primer Name	Sequence, 5′–3′	Template Volume	Per-Cycle Conditions (Denaturation/Primer Annealing/Extension)	Reference
*cytb*	First round	RNA_Cytb_737_F1	TTCCTACTTCCTTCATCTTHACTC	5 µL DNA	95 °C 30 s/55 °C 45 s/72 °C 2 min 30 s	This study
		Cytb_RNA_2550_R1	AGGAAACAGGGCTTTAACCA			
	Nested round	RNA_Cytb_1173_F2	ACTGATATTTTGGACAATTCTGACTTT	3 µL first-round product	95 °C 25 s/55 °C 45 s/72 °C 1 min 30 s	
		Cytb_RNA_2211_R2	CTTGGTCATGGTATTCTGGAATG			
18S rRNA	First round for *B. bovis* confirmation	Thei F1	AACCTGGTTGATCCTGCCAG	5 µL DNA	95 °C 30 s/50 °C 30 s/72 °C 30 s	Heidarpour Bami et al., 2009 [30]
		Thei R1	AAACCTTGTTACGACTTCTC			
	Nested round for *B. bovis* confirmation	Babesia _18SRNA_F	GTTTCTGMCCCATCAGCTTGAC	2 µL first-round product	95 °C 30 s/52 °C 30 s/72 °C 30 s	Hilpertshauser et al., 2006 [31]
		Babesia _18SRNA_R	CAAGACAAAAGTCTGCTTGAAAC			
18S rRNA	Single-round PCR for other *Babesia* spp.	Babesia _18SRNA_F	GTTTCTGMCCCATCAGCTTGAC	5 µL DNA	94 °C 30 s/58 °C 30 s/72 °C 45 s	
		Babesia _18SRNA_R	CAAGACAAAAGTCTGCTTGAAAC			
*cox1*	Single-round PCR	Babesia _Cox1_475-f1	CTGGAATAGCTAGTGCTATGAGTG	5 µL DNA	95 °C 25 s/60 °C 45 s/72 °C 1 min 20 s	This study
		Babesia _Cox1_475-f2	CTGGAATTGCAAGTGCAATGAGTG			
		Apicomplex _Cox1_1365_R1	TCCCCACATATCATCAGGATAATC			

***Notes: **cytb, *cytochrome b gene*; *18S rRNA, 18S ribosomal RNA gene;* cox1, *cytochrome c oxidase subunit I gene.

**Table 2:** Regional distribution of *Babesia* species identified by sequencing among 250 nested PCR-positive cattle samples from Kazakhstan.

Region	Number of Samples Sequenced, *n*	*Babesia major*, *n* (%)	*Babesia bovis*, *n* (%)	*Babesia bigemina*, *n* (%)	*Babesia occultans*, *n* (%)	*Babesia* spp. (Unidentified), *n* (%)
Abai	15	12 (80.0)	0 (0.0)	0 (0.0)	2 (13.3)	1 (6.7)
Akmola	10	0 (0.0)	0 (0.0)	0 (0.0)	8 (80.0)	2 (20.0)
Aktobe	9	0 (0.0)	0 (0.0)	0 (0.0)	7 (77.8)	2 (22.2)
Almaty	2	1 (50.0)	0 (0.0)	0 (0.0)	0 (0.0)	1 (50.0)
Atyrau	48	0 (0.0)	0 (0.0)	36 (75.0)	9 (18.8)	3 (6.3)
East Kazakhstan	0	NA	NA	NA	NA	NA
Jambyl	79	44 (55.7)	15 (19.0)	7 (8.9)	0 (0.0)	13 (16.5)
Jetisu	1	0 (0.0)	0 (0.0)	0 (0.0)	0 (0.0)	1 (100.0)
Karaganda	1	0 (0.0)	0 (0.0)	0 (0.0)	1 (100.0)	0 (0.0)
Kostanay	3	0 (0.0)	0 (0.0)	0 (0.0)	2 (66.7)	1 (33.3)
Kyzylorda	40	16 (40.0)	0 (0.0)	0 (0.0)	19 (47.5)	5 (12.5)
Mangystau	1	0 (0.0)	0 (0.0)	0 (0.0)	0 (0.0)	1 (100.0)
North Kazakhstan	0	NA	NA	NA	NA	NA
Pavlodar	0	NA	NA	NA	NA	NA
Turkistan	24	5 (20.8)	3 (12.5)	2 (8.3)	12 (50.0)	2 (8.3)
Ulytau	8	0 (0.0)	0 (0.0)	0 (0.0)	8 (100.0)	0 (0.0)
West Kazakhstan	9	0 (0.0)	0 (0.0)	0 (0.0)	6 (66.7)	3 (33.3)
Total	250	78 (31.2)	18 (7.2)	45 (18.0)	74 (29.6)	35 (14.0)

## Data Availability

The data presented in this study are openly available in NCBI and GenBank at https://www.ncbi.nlm.nih.gov/nuccore, reference number PZ544812–PZ544825 and PZ554284–PZ554324. All R codes for Figure 1 and Figure 2 and spatial analysis (Materials and Methods, Section 2.7) are available from https://github.com/asselakhmet/Spatial_analysis_Babesia_spp_Kazakhstan- (accessed on 11 June 2026).

## Supplementary Materials

The following supporting information can be downloaded at https://www.mdpi.com/article/10.3390/vetsci13080755/s1, Supplementary Figure S1: *Cytb* phylogenetic tree including all *Babesia* spp. sequences obtained in the present study; Supplementary Table S1: Names of sampled settlements, geographic coordinates (from Google Maps v11.65.00), distribution of *Babesia* spp. infection in adult cattle from 171 settlements across 17 provinces of Kazakhstan (sampling conducted in July–August 2022 and 2023); Supplementary Table S2: GenBank accession numbers of Babesia sequences generated in this study, including sample identifiers and target genes.

## Abbreviations

The following abbreviations are used in this manuscript:

TBD	Tick-borne diseases
GAM	Generalized additive model
*cytb*	Cytochrome b
PCR	Polymerase chain reaction
nPCR	Nested polymerase chain reaction
18S rRNA	18S ribosomal RNA
*cox1*	Cytochrome c oxidase subunit I
