# Supplementary material for: A Comprehensive Molecular Survey of Babesia spp. Genetic Diversity in Kazakhstan’s Cattle Populations
Source: Vet Sci. 2026 Jul 29;13(8):755. doi: 10.3390/vetsci13080755 (PMC13517960; doi:10.3390/vetsci13080755)
Supplement: Supplementary file 1 [file vetsci-13-00755-s001.zip › Supplementary Table S1-2.pdf]

Supplementary Table S1. Names of sampled settlements, geographic coordinates (from Google Maps), distribution of *Babesia* spp. infection in adult cattle from 171 settlements across 17 provinces of Kazakhstan (sampling conducted in July–August 2022 and 2023)

Province (oblast)	Administrative district	Settlement	Latitude	Longitude	N tested using PCR	N positive		*Babesia major*		*Babesia occultans*		*Babesia bigemina*		*Babesia bovis*		*Babesia* spp.	
						N pos.	% pos.	N pos.	% pos.[a]	N pos.	% pos.[a]	N pos.	% pos.[a]	N pos.	% pos.[a]	N pos.	% pos.[a]
**Turkistan**	**Subtotal province**				**790**	**24**	**3**	**5**	**21**	**12**	**50**	**2**	**8**	**3**	**13**	**2**	**8**
	Sayram	Karabulak	42.533632	69.755351	40	0	0										
		Karamurt	42.307077	69.969777	20	0	0										
		Mankent	42.440391	69.874677	20	0	0										
	Badam rural district	Badam settlement	42.381820	69.256509	40	6	15										
	Borjar	Uyalyzhar	42.431613	69.294784	40	0	0										
	Otrar	Aktobe	42.799580	68.713191	34	4	12										
		Talapty	42.861180	68.294127	40	3	8										
	Tulkibas	Abay	42.641091	70.114274	88	0	0										
	Kazygurt	Sharbulak	41.774444	69.490278	50	1	2										
	Tole Bi	Zhogargi Aksu	42.331369	70.067498	60	0	0										
	Sauran	Karashyk	43.357736	68.231624	43	0	0										
		Chernak	43.399573	68.016379	49	0	0										
	Saryagash	Han-Zhan	41.599341	69.347308	46	5	11										
		Zhilga	41.718253	69.013000	49	3	6										
	Baydibek	Kenestobe	43.036235	69.610353	46	0	0										
	Sozak	Syzgan	43.978105	68.545822	50	0	0										
		Sozak	44.138605	68.467201	25	2	8										
	Ordabasy	Temirlan	42.599137	69.256309	50	0	0										
**Jambyl**	**Subtotal province**				**879**	**79**	**9**	**44**	**56**	**0**	**0**	**7**	**9**	**15**	**19**	**13**	**16**
	Bayzak	Botamoynak rural district	42.978056	71.527500	50	14	28										
		Bayzak	42.977381	71.529450	50	1	2										
		Akchulak	43.005541	71.802885	30	0	0										
	Jambyl	Kolkaynar	43.059167	71.235556	48	0	0										
		Karatobinskoye	42.883057	71.284225	30	15	50										
	Turar Ryskulova	Terenonzek	42.978694	72.066562	50	0	0										
		Abay rural district	42.912313	72.800952	50	0	0										
		Akyrtobe	42.991073	72.101704	52	0	0										
	Talas	Berikkainar rural district	43.012481	70.580326	47	0	0										
		Koktal	43.266107	70.301813	47	0	0										

…continued.

Province (oblast)	Administrative district	Settlement	Latitude	Longitude	N tested using PCR	N positive		*Babesia major*		*Babesia occultans*		*Babesia bigemina*		*Babesia bovis*		*Babesia* spp.	
						N pos.	% pos.	N pos.	% pos.[a]	N pos.	% pos.[a]	N pos.	% pos.[a]	N pos.	% pos.[a]	N pos.	% pos.[a]
**Jambyl**	Talas	Karatau	43.175515	70.459523	28	0	0										
	Zhualy	Shinbulak	42.706488	70.650511	47	23	49										
		Kolbastau	42.607500	70.784167	48	15	31										
		Aksay	42.604679	70.762693	48	10	21										
		Bauyrjan Momyshuly rural district	42.619051	70.764328	49	0	0										
	Merki	Merki	42.872481	73.190139	205	1	0										
**Kyzylorda**	**Subtotal province**				**861**	**40**	**5**	**16**	**40**	**19**	**48**	**0**	**0**	**0**	**0**	**5**	**13**
	Karmakshy	3-d Internatsional	45.191667	64.128889	49	0	0										
		Taimbet Komekbaev rural district	44.838933	63.197639	52	1	2										
	Kyzylorda	Kyzylorda	44.842544	65.502563	50	0	0										
	Aral	Aral	46.797738	61.660792	50	1	2										
		Zhaksylysh rural district	46.915489	62.026956	26	0	0										
		Zhaksykylysh	46.824752	61.854119	25	0	0										
		Akkum rural district	46.942778	61.620000	25	0	0										
	Kazaly	Ayteke Bi	45.835934	62.148317	31	0	0										
		Sarykol rural district	45.732557	62.115942	47	12	26										
	Zhalagash	Zhalagash	45.080281	64.681333	44	0	0										
		Akkum rural	44.916664	64.712612	46	2	4										
	Zhanakorgan	Zhanakorgan	43.904444	67.251111	49	1	2										
		Zhamanbai batyr rural district	43.689722	67.766111	37	15	41										
		Aqqorghan rural district	43.592500	67.411111	45	0	0										
		Kirkense rural district	43.798333	67.124722	50	0	0										
	Shieli	Telekol rural district	44.259722	66.685556	49	1	2										

…continued.

Province (oblast)	Administrative district	Settlement	Latitude	Longitude	N tested using PCR	N positive		*Babesia major*		*Babesia occultans*		*Babesia bigemina*		*Babesia bovis*		*Babesia* spp.	
						N pos.	% pos.	N pos.	% pos.[a]	N pos.	% pos.[a]	N pos.	% pos.[a]	N pos.	% pos.[a]	N pos.	% pos.[a]
**Kyzylorda**	Shieli	Zhuantobe	44.267588	66.311218	44	3	7										
		Shieli	44.178889	66.732778	48	0	0										
	Syrdariya	Terenozek	45.051944	64.980556	47	1	2										
		Askar Tekmagambetov rural district	45.057148	65.052480	47	3	6										
**Mangystau**	**Subtotal province**				**200**	**1**	**1**	**0**	**0**	**0**	**0**	**0**	**0**	**0**	**0**	**1**	**100**
	Mangystau	Say-Otes	44.328530	53.533572	15	0	0										
		Shetpe	44.139155	52.162069	15	0	0										
		Zhyngyldy	44.198686	51.700201	15	0	0										
		Otpan rural district	44.149809	51.942377	5	0	0										
	Beyneu	Sam	45.236993	55.866650	13	0	0										
		Beyneu	45.322946	55.188862	17	0	0										
		Boranqul	46.208008	54.469042	13	0	0										
		Akjigit	45.039267	55.753112	7	0	0										
	Karakiya	Kuryk	43.178221	51.679423	26	1	4										
		Zhetybay	43.587320	52.104326	24	0	0										
	Munaily	Daulet	43.701879	51.321067	10	0	0										
		Mangystau	43.691561	51.305571	10	0	0										
		Kyzyltobe	43.692930	51.337874	10	0	0										
		Batyr	43.630290	51.408113	10	0	0										
		Bayandy	43.761104	51.306083	10	0	0										
**North Kazakhstan**	**Subtotal province**				**362**	**0**	**0**	**0**	**0**	**0**	**0**	**0**	**0**	**0**	**0**	**0**	**0**
	Zhambyl	Presnovka	54.671539	67.158203	50	0	0										
	Mamlyut	Shchuchye	55.153509	68.506134	10	0	0										
		Beloe	55.062823	68.473939	10	0	0										
		Krasnoznamennoe	54.977356	68.808948	10	0	0										
		Pokrovka	54.928831	68.636794	20	0	0										
		Ledenevo	54.699696	68.535069	20	0	0										
	Akkayin	Vlasovka	54.200965	69.600741	50	0	0										
		Kuchkova	54.146761	69.783296	4	0	0										
	Kyzylzhar	Semicol	54.600781	68.827183	50	0	0										
	Magzhan Zhumabaev	Zhastar	54.566944	70.212222	30	0	0										
		Sortomar	54.638351	70.167560	20	0	0										
	Esil	Amangeldy	54.177274	67.937977	61	0	0										
		Il'inka	54.201824	68.010444	10	0	0										
	Timiryazev	Dokuchaevo	53.914759	66.486964	15	0	0										
	Taiynsha	Karagash	53.630269	70.119024	2	0	0										

…continued.

Province (oblast)	Administrative district	Settlement	Latitude	Longitude	N tested using PCR	N positive		*Babesia major*		*Babesia occultans*		*Babesia bigemina*		*Babesia bovis*		*Babesia* spp.	
						N pos.	% pos.	N pos.	% pos.[a]	N pos.	% pos.[a]	N pos.	% pos.[a]	N pos.	% pos.[a]	N pos.	% pos.[a]
**Pavlodar**	**Subtotal province**				**300**	**0**	**0**	**0**	**0**	**0**	**0**	**0**	**0**	**0**	**0**	**0**	**0**
	Zhelezin	Tserkovnoye	53.603723	75.915951	50	0	0										
	Sharbaqty	Orlovka	52.576182	78.032966	25	0	0										
		Krasilovka	52.712993	77.792648	25	0	0										
	Akkuly	Caracala	51.722183	77.533026	50	0	0										
	Uspen	Uspenka	52.907129	77.419380	50	0	0										
	Ertis	Kosagash	53.114579	74.870959	50	0	0										
	Pavlodar	Pavlodar	52.285577	76.940722	50	0	0										
**West Kazakhstan**	**Subtotal province**				**200**	**9**	**5**	**0**	**0**	**6**	**67**	**0**	**0**	**0**	**0**	**3**	**33**
	Bokey Orda	Karakol	48.416667	46.616667	50	6	12										
	Zhanakala	Zhanazholsky	48.863829	49.892862	50	0	0										
	Akzhaik	Zhanabulak	50.314926	51.068254	50	3	6										
	Kaztal	Bostandyk	49.624068	48.881197	50	0	0										
**Aktobe**	**Subtotal province**				**190**	**9**	**5**	**0**	**0**	**7**	**78**	**0**	**0**	**0**	**0**	**2**	**22**
	Yrgyz	Zhaysanbai	48.039595	62.512135	50	0	0										
	Bayganin	Zharly	48.532598	55.591136	50	0	0										
	Temir	Jaksimaj rural district	49.203889	56.562500	50	2	4										
	Oiyl	Oiyl	49.074441	54.665629	40	7	18										
**Atyrau**	**Subtotal province**				**200**	**48**	**24**	**0**	**0**	**9**	**19**	**36**	**75**	**0**	**0**	**3**	**6**
	Zhylyoi	Kulsary	46.960532	54.006221	25	11	44										
		Aqqyztoghay	47.096629	54.375887	25	0	0										
	Inder	Inderbor	48.555353	51.742215	25	7	28										
		Koktogay	48.131198	51.534873	25	6	24										
	Isatay	Tushchikuduk	47.260314	51.067948	25	4	16										
		Rybachje	47.685336	50.839022	25	13	52										
	Kurmangazy	Primorye	46.550121	49.209460	50	7	14										
**Ulytau**	**Subtotal province**				**300**	**8**	**3**	**0**	**0**	**8**	**100**	**0**	**0**	**0**	**0**	**0**	**0**
	Ulytau	Mibulak	46.949910	68.287377	50	7	14										
	Zhezqazghan	Kengir	47.836945	67.618815	50	1	2										
		Zhezqazghan	47.799711	67.714090	50	0	0										
	Zhanaarka	Saken Seifullin rural district	48.903333	71.753333	50	0	0										
		Bidaik	48.725556	71.943889	50	0	0										
		Zhanaarka	48.685790	71.635155	50	0	0										

…continued.

Province (oblast)	Administrative district	Settlement	Latitude	Longitude	N tested using PCR	N positive		*Babesia major*		*Babesia occultans*		*Babesia bigemina*		*Babesia bovis*		*Babesia* spp.	
						N pos.	% pos.	N pos.	% pos.[a]	N pos.	% pos.[a]	N pos.	% pos.[a]	N pos.	% pos.[a]	N pos.	% pos.[a]
**Abai**	**Subtotal province**				**400**	**15**	**4**	**12**	**80**	**2**	**13**	**0**	**0**	**0**	**0**	**1**	**7**
	Ayagoz	Ayagoz	47.965266	80.436238	50	2	4										
	Beskaragay	Beskarahay	50.886001	79.480996	50	1	2										
	Kurchatov	Kurchatov	50.756325	78.548329	50	2	4										
	Zharma	Qalbatau	49.328084	81.573693	50	8	16										
	Abay	Kengirbay bi	48.902261	79.246401	50	1	2										
	Kokpekti	Ulken Boken	48.781692	82.694252	50	1	2										
	Borodulikha	Mikhaylichenkovo	50.643906	81.135253	50	0	0										
	Zhanasemey	Dostyk	50.454444	79.851389	50	0	0										
**Almaty**	**Subtotal province**				**248**	**2**	**1**	**1**	**50**	**0**	**0**	**0**	**0**	**0**	**0**	**1**	**50**
	Enbekshikazakh	Karashota	43.649992	78.183065	20	0	0										
		Asa rural district	43.526944	78.276111	10	0	0										
		Shelek rural district	43.599167	78.256667	10	0	0										
	Talgar	Nura	43.687611	77.193041	50	2	4										
	Zhambyl	Uzynagash	43.220925	76.314210	45	0	0										
		Sholak	44.037222	77.774722	33	0	0										
	Ile	Mezhdurecheнskoye	43.455160	76.723780	6	0	0										
		Akshi	43.991220	76.326822	26	0	0										
		Zhetygen	43.681261	77.116046	16	0	0										
		Karaoy	43.517111	76.833212	16	0	0										
		Baiserke	43.489634	77.040615	16	0	0										
**Jetisu**	**Subtotal province**				**200**	**1**	**1**	**0**	**0**	**0**	**0**	**0**	**0**	**0**	**0**	**1**	**100**
	Koksu	Jarlyozek	44.904599	78.054974	50	0	0										
	Aksu	Zhansugiruly	45.398587	79.503265	50	0	0										
	Eskeldi	Syrymbet	44.856519	78.682484	50	1	2										
	Kerbulak	Zhaynak	44.388171	78.585834	50	0	0										
**Kostanay**	**Subtotal province**				**428**	**3**	**1**	**0**	**0**	**2**	**67**	**0**	**0**	**0**	**0**	**1**	**33**
	Denisov	Pereleski	52.545399	62.074871	50	0	0										
	Kamysty	Kamysty	51.963814	61.779038	50	2	4										
	Karabalyk	Nadezhdinka	53.760294	63.743249	50	0	0										
	Fyodorov	Fyodorovka	53.636244	62.698023	50	1	2										
	Altynsarin	Tanabay	53.345962	64.578359	50	0	0										
	Amangeldi	Amangeldy	50.183089	65.186320	50	0	0										
	Zhangeldi	Torghay	49.632447	63.491837	50	0	0										
	Sarykol	Ostrovnoye	53.342672	65.477797	50	0	0										

…continued.

Province (oblast)	Administrative district	Settlement	Latitude	Longitude	N tested using PCR	N positive		*Babesia major*		*Babesia occultans*		*Babesia bigemina*		*Babesia bovis*		*Babesia* spp.	
						N pos.	% pos.	N pos.	% pos.[a]	N pos.	% pos.[a]	N pos.	% pos.[a]	N pos.	% pos.[a]	N pos.	% pos.[a]
**Kostanay**	Kostanay	Zarechnyi	53.2337054	63.736094	28	0	0										
**East Kazakhstan**	**Subtotal province**				**200**	**0**	**0**	**0**	**0**	**0**	**0**	**0**	**0**	**0**	**0**	**0**	
	Ulan	Aiyrtau	49.836250	82.265001	50	0	0										
		Gagarino	50.144975	81.848838	50	0	0										
	Glubokoye	Solnechnoe	50.050572	82.714572	100	0	0										
**Akmola**	**Subtotal province**				**200**	**10**	**5**	**0**	**0**	**8**	**80**	**0**	**0**	**0**	**0**	**2**	**20**
	Arshaly	Saryoba	51.203207	72.183154	200	10	5										
**Karaganda**	**Subtotal province**				**497**	**1**	**0**	**0**	**0**	**1**	**100**	**0**	**0**	**0**	**0**	**0**	
	Nura	Ahmet	50.635851	71.462418	50	0	0										
		Karaoy	50.256022	71.483187	50	0	0										
	Priozersk	Priozersk	46.028969	73.708027	50	0	0										
	Sarybel	Oshagandy	50.312947	72.734900	20	0	0										
		Molodezhnyy	50.726109	73.529028	15	0	0										
		Sunkar	50.381638	72.220192	15	0	0										
	Shet	Kayrakty	48.849579	73.603831	20	0	0										
		Kenshoki rural district	48.574444	73.983611	19	0	0										
		Akadyr	48.263632	72.856047	20	0	0										
		Akoi	49.021557	72.560097	20	0	0										
		Taldy	49.107591	73.748038	20	0	0										
	Karkaraly	Karakezen	49.595000	75.058333	49	1	2										
	Abai	Kulajgyr	49.425908	72.923348	50	0	0										
	Balkhash	Balkhash	46.843721	74.977301	20	0	0										
		Sayak	46.906506	77.356417	19	0	0										
		Gulshat	46.630004	74.355452	11	0	0										
	Aktogay	Nurken	48.107764	74.561076	7	0	0										
		Karamende Bi rural district	48.630000	75.028333	5	0	0										
		Zhidebay	47.811234	74.773941	9	0	0										
		Shabanbai Bi	48.402289	75.394254	8	0	0										
		Aktogay	48.316087	74.981362	8	0	0										
		Kyzylaray rural district	48.572778	75.487222	8	0	0										
		Saryterek	48.068076	75.338720	4	0	0										
**Total**					**6455**	**250**	**3,87**	**78**	**31**	**74**	**30**	**45**	**18**	**18**	**7**	**35**	**14**

[a]Percentage among positive cases by region
