# Supplementary material for: A Comprehensive Molecular Survey of Babesia spp. Genetic Diversity in Kazakhstan’s Cattle Populations
Source: Vet Sci. 2026 Jul 29;13(8):755. doi: 10.3390/vetsci13080755 (PMC13517960; doi:10.3390/vetsci13080755)
Supplement: Supplementary file 1 [file vetsci-13-00755-s001.zip › Supplementary Table S2-2.pdf]

Supplementary Table S2. GenBank accession numbers of *Babesia* sequences generated in this study, including sample identifiers and target genes.

Sample ID	Host species	Species	Gene	GenBank accession number
ATR-6608	cattle	Babesia occultans	*cytb*	PZ554293
ABAI-7283	cattle	Babesia occultans	*cytb*	PZ554294
KST-8826	cattle	Babesia occultans	*cytb*	PZ554295
ATR-6590	cattle	Babesia occultans	*cytb*	PZ554296
ULT-6734	cattle	Babesia occultans	*cytb*	PZ554297
WESTK-6150	cattle	Babesia occultans	*cytb*	PZ554298
WESTK-6040	cattle	Babesia occultans	*cytb*	PZ554299
ATR-6578	cattle	Babesia occultans	*cytb*	PZ554300
KZL-3784	cattle	Babesia occultans	*cytb*	PZ554301
TRK-1504	cattle	Babesia occultans	*cytb*	PZ554302
KZL-3782	cattle	Babesia occultans	*cytb*	PZ554303
KZL-4521	cattle	Babesia occultans	*cytb*	PZ554304
TRK-171	cattle	Babesia occultans	*cytb*	PZ554305
ULT-6737	cattle	Babesia occultans	*cytb*	PZ554306
TRK-1146	cattle	Babesia occultans	*cytb*	PZ554307
TRK-1118	cattle	Babesia occultans	*cytb*	PZ554308
TRK-177	cattle	Babesia occultans	*cytb*	PZ554309
KZL-4382	cattle	Babesia occultans	*cytb*	PZ554310
ULT-6756	cattle	Babesia occultans	*cytb*	PZ554311
AKM-9428	cattle	Babesia occultans	*cytb*	PZ554312
KST-8941	cattle	Babesia occultans	*cytb*	PZ554313
ULT-6810	cattle	Babesia occultans	*cytb*	PZ554314
ABAI-7186	cattle	Babesia major	*cytb*	PZ554316
ALM-7659	cattle	Babesia major	*cytb*	PZ554317
JMB-2868	cattle	Babesia major	*cytb*	PZ554318
JMB-2961	cattle	Babesia major	*cytb*	PZ554319
KZL-4124	cattle	Babesia major	*cytb*	PZ554320
JMB-2823	cattle	Babesia major	*cytb*	PZ554321
ATR-6558	cattle	Babesia bigemina	*cytb*	PZ554322
ATR-6427	cattle	Babesia bigemina	*cytb*	PZ554323
ATR-6520	cattle	Babesia bigemina	*cytb*	PZ554324
JMB-1634	cattle	Babesia bovis	*cytb*	PZ554315
ULT-6810	cattle	Babesia occultans	18S rRNA	PZ544820
AKM-9428	cattle	Babesia occultans	18S rRNA	PZ544821
KZL-3784	cattle	Babesia occultans	18S rRNA	PZ544822
TRK-1118	cattle	Babesia occultans	18S rRNA	PZ544823
JMB-2868	cattle	Babesia major	18S rRNA	PZ544812
JMB-1790	cattle	Babesia major	18S rRNA	PZ544813
JMB-2823	cattle	Babesia major	18S rRNA	PZ544814
KZL-4532	cattle	Babesia major	18S rRNA	PZ544815
JMB-2865	cattle	Babesia major	18S rRNA	PZ544816
ATR-6474	cattle	Babesia bigemina	18S rRNA	PZ544817
ATR-6497	cattle	Babesia bigemina	18S rRNA	PZ544818
ATR-6520	cattle	Babesia bigemina	18S rRNA	PZ544819
JMB-1634	cattle	Babesia bovis	18S rRNA	PZ544824
JMB-1636	cattle	Babesia bovis	18S rRNA	PZ544825
AKM-9428	cattle	Babesia occultans	*cox1*	PZ554284
ULT-6810	cattle	Babesia occultans	*cox1*	PZ554285
JMB-2823	cattle	Babesia major	*cox1*	PZ554286
JMB-2865	cattle	Babesia major	*cox1*	PZ554287
JMB-1790	cattle	Babesia major	*cox1*	PZ554288
JMB-2868	cattle	Babesia major	*cox1*	PZ554291
JMB-1634	cattle	Babesia bovis	*cox1*	PZ554289
JMB-1636	cattle	Babesia bovis	*cox1*	PZ554290
TRK-343	cattle	Babesia bovis	*cox1*	PZ554292

Abbreviations: ATR, Atyrau Region; ABAI, Abai Region; AKM, Akmola Region; ALM, Almaty Region; JMB, Jambyl Region; KST, Kostanay Region; KZL, Kyzylorda Region; TRK, Turkistan Region; ULT, Ulytau Region; WESTK, West Kazakhstan Region; *cytb*, cytochrome *b* gene; 18S rRNA, 18S ribosomal RNA gene; *cox1*, cytochrome *c* oxidase subunit I gene.
